# Supplementary material for: Patterns of Intron Gain and Loss in Fungi
Source: PLoS Biol. 2004 Nov 30;2(12):e422. doi: 10.1371/journal.pbio.0020422 (PMC532390; doi:10.1371/journal.pbio.0020422)
Supplement: Table S1 — Also available at http://genes.mit.edu/NielsenEtAl/. (4.3 MB ZIP). [file pbio.0020422.st001.zip › NielsenEtAl/html/902.html]

AN4536.1.NCU06249.1.MG05220.1.FG06970.1


```
 CLUSTAL W (1.82) Multiple Sequence Alignments - Introns Inserted


Sequence 1: NCU06249.1	1221 aa
Sequence 2: FG06970.1	1202 aa
Sequence 3: MG05220.1	1216 aa
Sequence 4: AN4536.1	1138 aa
Alignment Length: 1282 aa
Number Identitical Residues: 393 aa
Alignment Score (without introns) 21141


MG05220.1 	-MPPAADRGDNG1ATEF-HDE~DEDRGRRKLGPGPGLQIPKNRSTGNLAVIGETS--DRS
NCU06249.1	MLGIYGGNPENG1EERQRHDA~EGQARSASLSVSPALHLPKNKSTGNLSVTNDSS--EKA
FG06970.1 	MIQMHGGNNDHG1AES--HTG~EDQRGREKFPM--ALSLPKNRSTGNLAAARNSSTQDRS
AN4536.1  	----------MG~AASYNPDG1EPSPLSSPLGR-------LSESPEDESFMTPLSDDQTN
          	           *          : .     :          ..*. : :     * .:  

MG05220.1 	RIRFSFDAG---SAEADLDLLTR2SPIM-DHDHGLGLSGLRRIRQQHS-MRQPTLPSNP-
NCU06249.1	RLRFSFDAG---PAAVEYDAMAR2SSVMTDHDHGLGLSGLRRIRQQPLPLRTGTLPSAPG
FG06970.1 	RARMSLDASAAAAAAADLEAIAR2SPILTDHEHGLGLSGLRRIRQQRPPSRNPTLPGSRA
AN4536.1  	SSKYSVENT-----SAGFDVLPR2SPLLEQHEHGLGPSGLDRIRSQPP-SRVFTLPNMST
          	  : *.:        .  : :.* *.:: :*:**** *** ***.*    *  ***.   

MG05220.1 	SSRSPSVARLPRSASMTLMPPPRRQGSNISLSPGPASP--TFTEDLSRFPSESLHSFSFA
NCU06249.1	SRKPSFGSAITRSASLTTMLD--VHSNSIPLSSVPNSPSIAFTEDLSRFPSESLHSFSFA
FG06970.1 	SSRSPSVAALSRSTSMSAMAA--SVG-SLSLTGGPATP--SFADDLSRFPSESLHSFSFA
AN4536.1  	SSIG-ALSPRTLSPSPRSLSS--SRANSMAVSSSQDIN---TLEDLHRFPSESLHSFSFA
          	*      :  . *.*   :      ...:.::           :** *************

MG05220.1 	NQSEELIHSRQNVLKRSIEFMRDHMGWAAAN-STDPAMASAQARFSGDKEAQSMLELLAR
NCU06249.1	HQSEDLIHNRQNVLKRSIEFMKDRMGWSVASNTANAALASVQARITGDVETQNVLDLLAR
FG06970.1 	NQSEDFLHNRQNVLKRSIEFMKDHMGLPMSS--SQAALASAQARVSGDVETQNMLDLLAQ
AN4536.1  	QQSEELLHTRQNILKRSIDFMRDRFKWGPGS---TTGVASPPNRMRGDTDTQAMVDLMSQ
          	:***:::*.***:*****:**:*::    ..    ..:**   *. ** ::* :::*:::

MG05220.1 	AQVVAAGNLPNPGSTLPYGPLTGPPDVSGVNVFDRGFV-QRTSS-------PEPLEQSPD
NCU06249.1	AQLVGAGSLPNGDVTLSTGPLTGPAELSGENIFDKQFMVPRSASPEPLEDDESIAPESPA
FG06970.1 	AQLIRAGNLPNPDESYTPAPLTGPAEVSQENVFDKNFN-PRTSS----TDLTSPHQTSPP
AN4536.1  	SSIFGAS----------FGPMTGPADLGSDNVFDRTFT-----------DLQRPLPEAKD
          	:.:. *.           .*:***.::.  *:**: *            .       :  

MG05220.1 	FPEGLPGQEPKRSGSTSR--GTSDSAESSRTPTNESGASAQTAQTSPPISRRPSVLQRTL
NCU06249.1	SQKTIGATKSQKETDTSRPAKFSLGNDSEHSSRSTTNESGTTAQTSPSTSLRPGFLKRTM
FG06970.1 	PRRSR--VETTRRSNNLEPVAHETGRESDQSPTATNESQSASRKSAPARPKLT--LKRTM
AN4536.1  	FGQPP----------------SQLPAQPHLTSSQQLPHERRGLKSAPASRRVS--LKRTF
          	  .                   .   :.  :.      .    :::*.    .  *:**:

MG05220.1 	TDTRHVQIEQKLVTAISKPYAAS-TGVDPILSPTTVESFKAAGGNFASSVPQSAPHGHAS
NCU06249.1	TDTEHAQMQQKLMDVMATPFLAVDSGPQDHLPSPARQSFSSVSSQ--PPAVSSAVHGHST
FG06970.1 	TDITAVSAQDKLIDTVSQPFLTGQPIYQEPLSSASLTQLPSATTANFPTQLGSSVHGHTN
AN4536.1  	TDVSSAIPQRQLIEPLAQPYPTADPFSP--LGTPIIG----------SVFPTPALHTHSS
          	**   .  : :*:  :: *: : ..     * ..             .    .: * *:.

MG05220.1 	RWVPAAQAIFTTEAKAPWTILVANDLACLVFGVTKAEVRKMGILEVVQDDRRAWLANRLQ
NCU06249.1	RWAPAAQAIFTTESKPPWTILAANDLACLLFGVTKAEVRKMGILEVVQEERRAWLERKLL
FG06970.1 	RWVPAAQAIFTTEVKPPWTIIAANDLACLVFGVTKAEVRKMGILEVVQEERRSWLERKLL
AN4536.1  	KWNPVSQAVFRTESKAPWTILAANDLSCLVFGVTQAEVRKLSILEVVQEDRRQWLESKLR
          	:* *.:**:* ** *.****:.****:**:****:*****:.******::** **  :* 

MG05220.1 	GNDVDEVGDFAESESSSPEPLSRVASNSLLGSRGGGITAHLLSKPNSRAKASAKTQAAKK
NCU06249.1	QGSSDDAGDGSESEIAKP--VSNPPMSSILGSRGGGITAKLLSKPNSRNQPP-KSNAGRR
FG06970.1 	NQPDEYSEGQTPSGQVTP--AAS-AASALLNGRSG-ITAQLLSKPNSRTQP--RSYTQRR
AN4536.1  	NPTTDAAAKARPSPDKSR---MKPAVPKSMG-MGNGVTAQLLSKPPSRARPS------RR
          	    :       *   .       .    :.  ...:**:***** ** :..      ::

MG05220.1 	PKTNHDSESRLYKTAS-----SQGGRSNGVLLCGDVVPIK~KRNGATGSASLWVKEKHGG
NCU06249.1	AATIHNGDPKPPKPG---QGHHPATQSRGVLLCGDVVPIQ0KRNGATGSASLWVKEKKVG
FG06970.1 	AQTVHSGDPNPPKVGGGGHGHNQTNSSRGVLLCGDVVPIQ~KRNGATGSASLWVKEKKVG
AN4536.1  	AQTDDGYGSSVYTAR---QLNHPATKSRGVLLCGDVVPIQ~KRNGAKGSASIWVMEKRGG
          	. * ..  .   .             *.***********: *****.****:** **: *

MG05220.1 	LIWVVEEISEDTATVTLKEESIVEKLDGAVDAIWGGADARPGVDVGKLIPRIPRQGIDPR
NCU06249.1	LIWVLEEIHEDVAYLTLDEEGVVLKLSGALGPIWGDENLKPGMDVGKLIPRIPRQGFDPR
FG06970.1 	LIWVLEEIHEDVAYITLDEEGIVQQVSGSTAPIWGFESISSGFDIARLIPRIPRQGIDPN
AN4536.1  	LIWVLEEIQEDVAYIDCDESWNITAIRGAAGRIWGHGMTEIGQHLSKLLPQLPPSSLKAP
          	****:*** **.* :  .*.  :  : *:   ***      * .:.:*:*::* ..:.. 

MG05220.1 	SGELDYAQINKKRFYTCRYSDTVNIPATVEQIRGKTELKVSSFPHIAGIIVVNTHDLRIK
NCU06249.1	TGEIDYAQIAKRRYYTCRNSDRINIPATIDQVRGQSELRVASFPHIAGIVVVCPQTFTIK
FG06970.1 	TGEIDFAQATRRRYFTCPHSPHVNIPCTIEQTRGKLELRVSTFPHMAGIIVVEPESLKVR
AN4536.1  	-AEKGLSEVADIKYFAASTSGPICTPVTVSKDERSRSLRVSSYPHVAGIMVLSSSSLKIV
          	 .* . ::    ::::.  *  :  * *:.: . . .*:*:::**:***:*: .  : : 

MG05220.1 	SSNSVFSGALFGYEKPDGMSITTLIPSFEKMLDVVTQDN-WSLEEAVVVPEQSFRQAYAI
NCU06249.1	SSNSVFCGALFGHEKPDGLPISHLVPDFAGVIRILKDEDGIDLVDGIVIPEHSFRKAAAF
FG06970.1 	SSNSAFCGALFGFERADGMSINALIPEFDRILESLIEEDGIQLLDGMVVPEHRFRKASAF
AN4536.1  	SSNSVFSSVLFGYERPEGLDITDLIPGFDDFLYVISEEENVPLVDGVVIPELSFRRARTL
          	****.*...***.*:.:*: *. *:* *  .:  : :::.  * :.:*:**  **:* ::

MG05220.1 	VGLREGNLDAPTSSMRPYGVPAKHRDGSEVKIDIQMRVVQSESDKNHVIPEDAAVEDYSD
NCU06249.1	LALKEGRPDAATSLLRPEGLPAKHRDGSELKIDIQMRVVKSE--KQTQISDQAIAEESED
FG06970.1 	LALKENRPDAASAFLHPAGLSAKHRDGSDLKVDVQMRVVKSE--KKTLVVNETVAEGSDE
AN4536.1  	SMLRDGKANVASVFLEPAGITAKHRDGSTIAVDVQLRVVKSG----SIFPKQREKKGDIE
          	  *::.. :..:  :.* *:.******* : :*:*:***:*       . .:   :   :

MG05220.1 	EASRRLSNAS----MPQAEMVYALWITYSRHLHASQSHAGGAASPLLSRRETQMHQPTPG
NCU06249.1	EESVSGADMTDSFTVSQSELVYALWITYSRHLHATKSNPG-FASPLLSGTATPLHQPSPG
FG06970.1 	DNAIGGDDAT--FEVTHSEIVYALWVTYSRHLHGTQPHLA-IETPTRSGALTPLHQPSPG
AN4536.1  	EEADDRPEGA----VTVTELVYALWITYSRNIHSHGSPVR-PPTPHEVSSSATSTPETPT
          	: :    : :    :. :*:*****:****::*.  .      :*      :     :* 

MG05220.1 	QTPAHTPP-LEGDS------ETEEPKKDTTISKFTRQIKEVAREAAAKLSSVTSSKPKEQ
NCU06249.1	QTPAHTPPKMDSDSSSESECETPSQRRESTTSLITRQLKEVAMTAAAKLTGTPKA---AE
FG06970.1 	QTPAHTPLEIPSDD------ETPRKNVMNAASSLSKHLKDAAIHAAAKITGQQTK---PK
AN4536.1  	SKPSPGTPTGPRIT------VPEGMKSRIPTSTLSQQLSEAASEPLT-----------DK
          	..*:  .              .   .   . * :::::.:.*  . :            :

MG05220.1 	PTAMVADTKEVVKETTAEAPAKPRKKTIDDFVILEEMGQGAYGQVKLAKHKET-GKTVVL
NCU06249.1	PKTEDIQTPKTVET--------PKKKTIDDFVILEDMGQGAYGQVKLARYKNS-GKKCVL
FG06970.1 	PE-EAPPVPKVVEP--------PHKKTINDYTILEEMGQGAYGQVKLARHKES-GKKIVL
AN4536.1  	PVQLVPEVKPANNK------EAPKKRSISDYVILEEMGQGAYGEVKLARLKKVPSKKVVL
          	*      .  . :         *:*::*.*:.***:*******:****: *: ..*. **

MG05220.1 	KYVIKERILVDTWTRDRRLGTVPSEIHILDFLGRNDLRHPNIIDMEGFFEDKVNYYIEME
NCU06249.1	KYVTKKRILVDTWTRDRRLGTVPLEIHVLDFLRREGFRHPNIVEMEDFFEDSVNYYIEMV
FG06970.1 	KYVTKRRILVDTWTRDRKLGTVPLEVHVLEYLRRPELRHPNIVEMQGFFEDDVNYYIEMV
AN4536.1  	KYVTKKRILVDTWTRDRRLGTVPLEIHVLDYLRRDGLKHPNIVEMEGFFEDDINYYIEML
          	*** *.***********:***** *:*:*::* *  ::****::*:.****.:****** 

MG05220.1 	PHGLPGMDLFDYIELRKNMSEDEARRIFVQLARAVHHLHIKAKIVHRDIKDENVILDGGG
NCU06249.1	PHGLPGMDLFDYIELRTNMDEQESRSIFVQVARAIHHLHTKALVVHRDIKDENVILDGEG
FG06970.1 	PHGLPGMDLFDYIELRTNMDEGECRSIFVQVANAIHHLHTEAKVVHRDIKDENVILDGEG
AN4536.1  	PHGLPGMDLFDYIELKTNMDEQECRNIFEQVVSAIHHLHTKALVVHRDIKDENVILDGEG
          	***************:.**.* *.* ** *:. *:**** :* :************** *

MG05220.1 	HVKLIDFGSAAYIRKGPFN0TMRPPR-----------------Y~WVEGDTMARPKTCGR
NCU06249.1	RIKLIDFGSAAYIKSGPFD~VFVGTI-----------------D1YAAPEVLAGKPYGGK
FG06970.1 	NIKLIDFGSAAYIKSGPFD~VFVGTI-----------------D1YAAPEVLAGKPYRGM
AN4536.1  	RIKLIDFGSAAYIKNGPFD~VFVGTIGKVSAAIFLGQKLTILLD~YAAPEVLQGKSYRGK
          	.:***********:.***: .:  . .. :::   ... :     :.  :.:      * 

MG05220.1 	S------ASYFMTIVYKENPYYCLDEIVDHELRIPWI-LSDENIDLIKGMLNRNVEERLN
NCU06249.1	EQDVWALGILLYTIIYKENPFYSIDEIMDRDLRVPYV-ISDESIDLIRKMLDRDVSQRST
FG06970.1 	EQDVWALGILLYTIIYKENPFYSIDEIMDRDLRIPYV-LSEESIDLIRCMLDRDVAKRYD
AN4536.1  	EQDIWALGILLYTIVYKENPFYNIDEILDHPLRVPFIPFSEDCIDLIRRMLDRDVDNRLT
          	...  : .  : **:*****:* :***:*: **:*::.:*:: ****: **:*:* :*  

MG05220.1 	IEQVLEHPWCKAAGDDFDGLHAIPGPSN
NCU06249.1	IEEVLAHPWCAMADD-------------
FG06970.1 	INQVLEHPWCQAANDE------------
AN4536.1  	IGEVMEHPWMVDG---------------
          	* :*: ***   .
```
